# Nanomedicine-based strategies to improve treatment of cutaneous leishmaniasis

**DOI:** 10.1098/rsos.220058

**Published:** 2022-06-15

**Authors:** Nowsheen Goonoo, Marie Andrea Laetitia Huët, Itisha Chummun, Nancy Karuri, Kingsley Badu, Fanny Gimié, Jonas Bergrath, Margit Schulze, Mareike Müller, Archana Bhaw-Luximon

**Affiliations:** ^1^ Biomaterials, Drug Delivery and Nanotechnology Unit, Center for Biomedical and Biomaterials Research, University of Mauritius, Réduit 80837, Mauritius; ^2^ Department of Chemical Engineering, Dedan Kimathi University of Technology, Private Bag 10143 – Dedan Kimathi, Nyeri, Kenya; ^3^ Vector-borne Infectious Disease Group, Theoretical and Applied Biology, Kwame Nkrumah University of Science and Technology, Kumasi, Ghana; ^4^ Animalerie, Plateforme de recherche CYROI, 2 rue Maxime Rivière, 97490 Sainte Clotilde, Ile de La Réunion, France; ^5^ Department of Natural Sciences, University of Applied Sciences Bonn-Rhein-Sieg, Heisenbergstrasse 16, D-53359 Rheinbach, Germany; ^6^ Physical Chemistry I & Research Center of Micro- and Nanochemistry and (Bio)Technology (Cμ), Department of Chemistry and Biology, University of Siegen, Adolf-Reichwein-Strasse 2, 57076 Siegen, Germany

**Keywords:** leishmaniasis, drug delivery, nanomedicine, scaffolds, tissue engineering

## Abstract

Nanomedicine strategies were first adapted and successfully translated to clinical application for diseases, such as cancer and diabetes. These strategies would no doubt benefit unmet diseases needs as in the case of leishmaniasis. The latter causes skin sores in the cutaneous form and affects internal organs in the visceral form. Treatment of cutaneous leishmaniasis (CL) aims at accelerating wound healing, reducing scarring and cosmetic morbidity, preventing parasite transmission and relapse. Unfortunately, available treatments show only suboptimal effectiveness and none of them were designed specifically for this disease condition. Tissue regeneration using nano-based devices coupled with drug delivery are currently being used in clinic to address diabetic wounds. Thus, in this review, we analyse the current treatment options and attempt to critically analyse the use of nanomedicine-based strategies to address CL wounds in view of achieving scarless wound healing, targeting secondary bacterial infection and lowering drug toxicity.

## 1. Introduction

**Figure 1.**
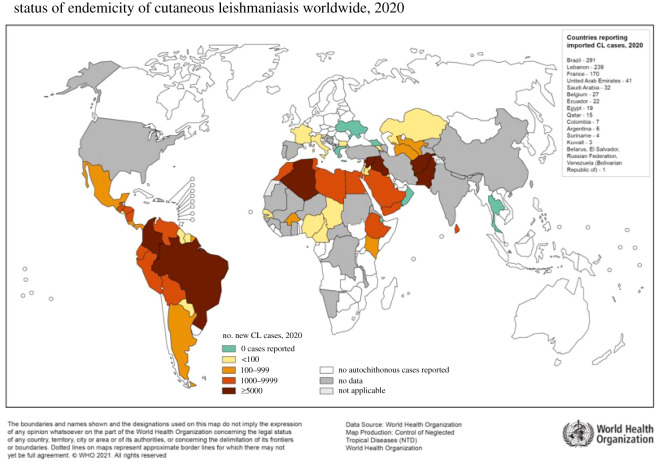
Map showing distribution of CL around the world. Reproduced with permission from [2], under the World Health Organization (WHO) copyright policy 2021.

Leishmaniasis is a protozoan parasitic disease found in parts of the tropics, subtropics and southern Europe. It is transmitted to humans and animals by the bite of phlebotomine female sandflies which are the vectors of at least 30 species of the genus *Leishmania* [1] (figure 1). The most common forms are cutaneous leishmaniasis (CL), which causes skin sores, and visceral leishmaniasis, which affects several internal organs.

Leishmaniasis currently affects around 12 million people in 98 countries with a widespread distribution in the developing world. For CL, estimates of the number of new cases per year have ranged from approximately 700 000 to 1.2 million or more [2]. Chronic forms of leishmaniasis include diffuse cutaneous leishmaniasis (DCL), mucosal leishmaniasis (ML) and leishmaniasis recidivans. The skin sores of CL usually heal on their own, even without treatment. But this can take months or even years, and the sores leave prominent scars which often lead to social stigmatization. *Leishmania* parasites causing CL can be divided into Old World species which include *Leishmania major, Leishmania tropica* and *Leishmania aethiopica*, common around the Mediterranean Basin, the Middle East, the Horn of Africa, or the Indian subcontinent; and New World species, such as *Leishmania amazonensis, Leishmania mexicana, Leishmania braziliensis* and *Leishmania guyanensis*, which are endemic to Central and South America [3].

**Figure 2.**
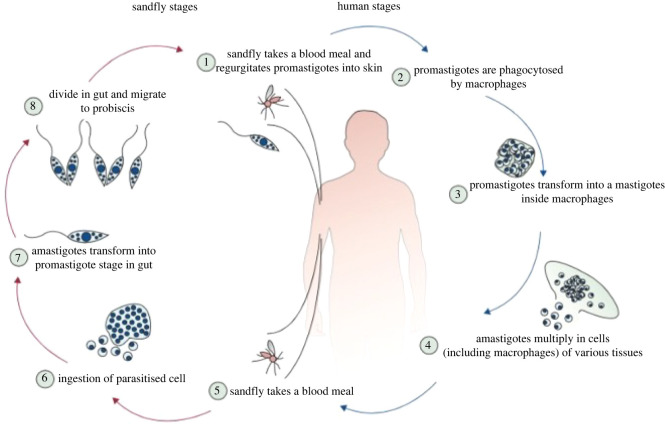
Transmission of leishmania parasites via the sandfly vector or the human host. Reproduced with permission from [1].

Species-based identification of the parasite is critical for the disease prognosis [4]. Sandflies, humans and animals can act as host in the transmission of leishmaniasis. In sandflies, the parasites proliferate in the promastigote (flagellate) distinct forms in the latter's hind gut. Procyclic promastigotes multiply in the bloodmeal within the mid-gut transforming into nectomonad promastigotes which migrate to the anterior mid-gut, transform into lectomonads in a second growth cycle, after which they differentiate into metacyclic promastigotes for onward transmission into vertebrates [5]. In vertebrates, macrophages located in the dermis phagocytose the extracellular promastigotes transforming them into intracellular non-flagellated amastigotes [6] (figure 2). Emerging amastigotes emanating from lysed macrophages are internalized by neutrophils and dendritic cells (DC). This marks the beginning of inflammation after several weeks when there is influx of neutrophils, followed by inflammatory macrophages. At this stage, clinically apparent lesions are observed. In the end, T-cell-derived IFN*γ* effects lesion resolution by initiating parasite killing. Subsequently, DC prime and activate antigen-specific T cells, thereby eliciting the adaptive immune response against *Leishmania* [7]. In the absence of any treatment, this process can take up to 18 months.

The development of the disease follows a complex pathway involving interactions between factors triggered by the host's innate and acquired immune responses. Inflammatory responses determine disease expression, i.e. symptomless or subclinical infection, self-healing CL, or chronic leishmaniasis. In addition, the resolution of the disease is controlled by cell-mediated responses rather than the humoral immune response and there is strong correlation between T-cell activation and disease outcome [1] that shows also clear differences between males and females, with males exhibiting a higher risk of the New World CL, that seems to be driven by a sex-dependent differential immune response [8].

It is established that sandfly saliva, similar to other haematophagous insects, holds several bioactive molecules which have anti-inflammatory and immunomodulatory functions. These facilitate blood feeding as well as potentiate the parasite infection and modulate host immune response [9–12]. The microenvironment of naive macrophages (M0) provides signals to activate the development of either ‘classically activated’ (M1) by Th1 lymphocytes with a variety of cytokines crucial in the killing of *Leishmania* through an oxidative burst [13,14], or ‘alternatively activated’ (M2) via Th2 lymphocytes, which produce IL-4 and IL-13 cytokines, inducing the M2 phenotype characterized by polyamine biosynthesis known to support the growth of *Leishmania* parasite within macrophages and thus overt disease [14,15]. Sandfly saliva can modify the microenvironment of naive macrophages and produce signals that favour alternatively activated macrophage (M2) profile in a variety of ways. Sandfly saliva induces IL-10 to elicit a regulatory response which is linked to the activation of a Th2 through the upsurge in IL-4 and IL-6 synthesis [16–20].

To date, there is no vaccine or safe drug to inhibit proliferation of the parasite. The absence of microscopy at basic healthcare facilities in many African countries poses a major challenge in leishmaniasis diagnosis as the presence of *Leishmania* amastigotes in clinical specimens is conducted using direct microscopic examination or molecular analysis. The *Leishmania* parasite has a complex life cycle and one of its developmental forms, namely the amastigote, resides within phagocytes, which explains the challenge of targeting the parasites with specific drugs [21]. Additional challenges include side effects caused by toxic drugs, non-responsiveness to treatment due to drug-resistant strains and poor compliance to treatment. In addition, it is very difficult to find a drug that will be effective against all forms of CL. The level of inflammation response further complicates treatment.

Nanomedicine forms part of modern medicine strategies to address challenges of both infectious diseases such as tuberculosis [22] and non-infectious diseases such as cancer [23]. Tissue regeneration using nano-based devices coupled with drug delivery are currently being used in clinics to address diabetic wounds [24]. Thus, in this review, we give an overview of the current treatment options and attempt to critically analyse the use of nanomedicine-based strategies to address CL wounds, which includes tissue regeneration, infection management and addressing inflammatory response.

## 2. Current diagnosis and treatment options for cutaneous leishmaniasis

### 2.1. Diagnostic tools

To date, there is no single reference test to detect CL, but observation of amastigotes in clinical specimen samples confirms the diagnosis. Highly sensitive molecular methods such as PCR are particularly helpful in mucosal lesions where the parasitic load is low. They further allow for species identification. Recently developed real-time kinetoplast DNA PCR (KDNA PCR) assays such as loop-mediated isothermal amplification (LAMP) technique displayed 98% sensitivity on 40 CL patients [25]. Emerging immunological tests involving the use of chemiluminescent ELISA to quantify anti-α-galactosyl antibodies are up to nine times higher in people with *L. tropica* or *L. major* infections than in healthy people [26].

An immunochromatographic rapid diagnostic test (IC-RDT), CL Detect™ kit (InBios International Inc., USA), was recently developed to detect amastigotes from CL skin lesions. The kit works by detecting peroxidioxin, produced by *Leishmania* promastigotes and amastigote from cell lysate. The sensitivity of this method varied among species and geographical strains. It was reported that the IC-RDT kit showed high sensitivity (100%) and specificity (96%) to *L. major* in Tunisia [27] but demonstrated poor efficiency to detect *L. dovani* amastigotes within CL lesions from patients in Sri Lanka (sensitivity 36%). This diagnostic method is highly dependent on parasite count and expression of peroxidioxin antigen which may vary between *Leishmania* species [28].

### 2.2. Treatment options

**Figure 3.**
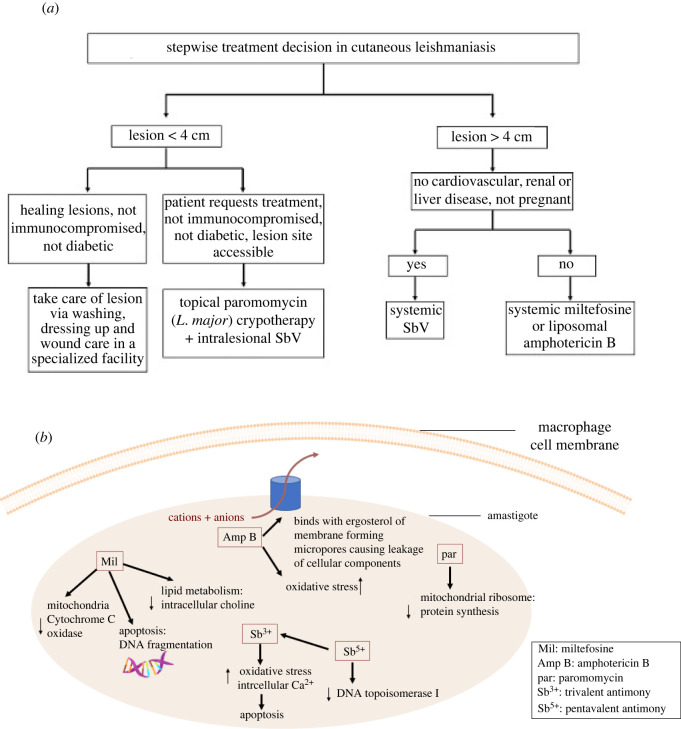
(*a*) Stepwise decision regarding treatment of CL and (*b*) mechanism of action of common anti-leishmanial drugs.

Available treatments for CL show only suboptimal effectiveness and none of them were designed specifically for CL (figure 3*a*). The first-line treatment approach remains pentavalent antimonial drugs (i.e. sodium stibogluconate or meglumine antimonate) at 20 mg kg^−1^ per day for 20–28 consecutive days, which have high toxicity and requires patient hospitalization. Treatment recommendation for CL by WHO depends on the parasite species, geographical location and the clinical manifestations. No treatment is recommended for leishmaniasis caused by *L. mexicana* or *L. major* [29]. Second-line treatment includes amphotericin B (AMB) deoxycholate (Fungizone^®^, 30 days with 1 mg kg^−1^), liposomal amphotericin B (AmBisome^®^, single-dose 10 mg kg^−1^), pentamidine (Pentam^®^, 3–5 days with 4 mg kg^−1^), miltefosine (MILT) (Impavido^®^, 28 days with 1.5–2.5 mg kg^−1^ d^−1^) and paromomycin (PRM) (Humatin^®^, 21 days with 15 mg kg^−1^ d^−1^) and are advised for complicated cases, non-responders to topical treatments, immunocompromised patients and for areas with high possibility of disease progression to mucosal *Leishmania* [29]. Amphotericin B is very effective but presents toxic effects when injected in the deoxycholate form [21]. FDA-approved lipid formulation of amphotericin B, AmBisome, is better tolerated than conventional amphotericin B but its high cost limits its use [30]. Local therapy such as thermotherapy (use of heat) and cryotherapy (application of subzero temperatures) have been used due to the thermosensitivity of the parasites [31]. Thermotherapy may be co-administered with infrared light, laser or direct electrical stimulation. The mechanisms of action of anti-leishmanial drugs have been detailed in several reviews [32,33] (figure 3*b*). Trivalent (SbIII) and pentavalent (SbV) forms of antimony inhibit trypanothione reductase and topoisomerase I enzymes. Amphotericin B acts on both promastigote and amastigote stages of the parasite by binding to their cell wall. In addition to affecting the membrane potential of the mitochondria, miltefosine and paromomycin inhibit cytochrome-c oxidase and protein synthesis respectively causing parasite death [32]. As a result of toxic side effects associated with these drugs such as nausea, vomiting, diarrhea, increased blood sugar, etc., careful monitoring of the patients is required.

The duration of treatment for persistent, multiple and large lesions lasts in general more than six months. However, treatment is only successful in patients with immunocompetent systems. Relapse is common in immunocompromised patients [2].

#### 2.2.1. Need for personalized treatment

**Figure 4.**
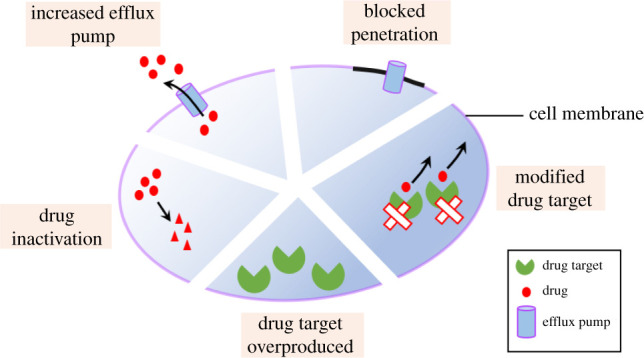
Molecular mechanisms involved in drug resistance in infected macrophages.

Therapeutic outcomes depend on (i) host factors (immune system, age, sex, compliance), (ii) parasite factors (species, strain, virulence, *Leishmania* RNA virus, resistance gene involved), (iii) drug-related factors (dosage, pharmacodynamics, pharmacokinetics, preservation, etc.), and (iv) drug resistance. Drug resistance (DR) is a major issue and occurs as a result of genetic mutations which reduce the parasite's response towards a given drug through decreased uptake of the drug by macrophages. Several mechanisms have been proposed for DR (figure 4) and targeting them may be a viable strategy to overcome DR. For instance, once inside immune cells, drugs may be inactivated, removed or relocated into vacuoles. Prodrugs require activation, and following DR, this important activation process is suppressed. A third mechanism of DR consists of changes in drug/target interaction due to modifications or higher number of target molecules [34]. Hence, a personalized medicine approach has enormous potential to improve treatment.

#### 2.2.2. Targeted approach

Following technological advances in clinical research, the availability of complete genome sequence of *Leishmania* and better understanding of the biological pathways of the *Leishmania* species, a number of potential therapeutic targets have been identified (table 1). They may eventually pave the way towards the development of new or repurposed drugs for the treatment of CL.

**Table 1.** Summary of main therapeutic targets, potential drugs (active agent) and their corresponding mode of action.

therapeutic target	agent	mode of action
glycolysis [35]	inhibitors of enzyme transport in glycolysis	arrest of glycolytic influx and killing of parasite
fatty acid and sterol metabolism [36,37]	fatty acyl-CoA ligase sterols such as ergosterol and 24-methyl sterol as well as enzymes involved in sterol biosynthesis including squalene synthase	disrupts cellular homeostasis of lipids
polyamine metabolism [38–42]	ornithine decarboxylase, trypanothione synthetase, trypanothione reductase, deoxyhypusine synthase and deoxyhypusine hydroxylase.	interferes with cell survival, growth and proliferation
proteasome and cell cycle [43,44]	inhibitors targeting cyclin-dependent kinases, histone acetyl transferase and histone deacetylases, SIR2 deacetylase	disrupts cell cycle
ER-mediated pathway of protein processing [45–47]	signal peptide peptidase (SPP) and agents leading to overexpression of calreticulin, BiP and protein disulfide isomerase (PDI)	interferes with the folding of proteins in endoplasmic reticulum and with their transport through the golgi for secretion outside the cell

**Figure 5.**
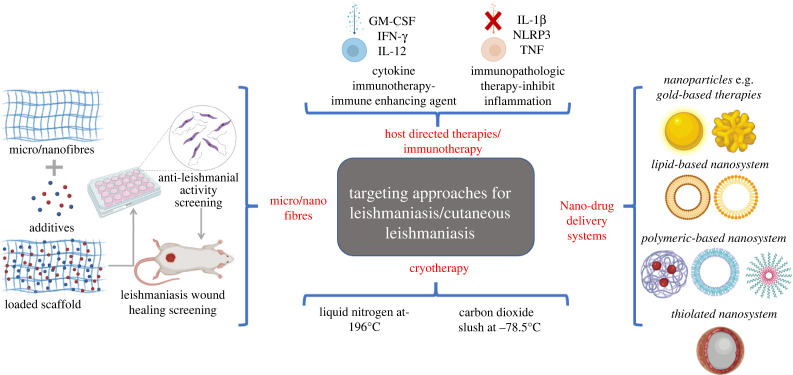
Targeted approaches for the treatment of CL.

The key to host-directed therapy is to define the mechanism that would promote immune protection and mediate immunopathological responses associated with the disease. Cytokines such as IFN-y, IL-12 or IL4 have been shown to control parasite growth and promote healing by increasing protective immunity [48]. However, while cytokines such TNF-a and IL-1ß are essential for macrophage activation, excess levels of these cytokines contribute to chronic inflammation. A number of inhibitors designed to block their pathway have been investigated. The pathological role of CD8T cells which have shown to produce little IFN-y and become cytolytic in lesions contributing to metastasis in leishmaniasis patients have been studied. CD8T cell-mediated disease could be blocked by inhibitors of NLRP3 and IL-1ß [48]. Figure 5 illustrates various targeting approaches in the treatment of leishmaniasis/CL.

Nano-drug delivery systems (nano-DDS) such as niosomes, liposomes, transfersomes and polymeric nano-DDS have been designed for topical and oral delivery in CL. Parasitic resistance can be avoided by using drug-loaded surface-modified nano-DDS systems (mannosylated or thiolated) [49]. Nanoparticles such as gold complexes have emerged as anti-leishmanial agents whereby gold-based drugs exhibited immunomodulation activity, thioredoxin reductase inhibition and redox imbalance [50]. Another targeting approach for CL is host-directed therapies aiming at modulating the severity of CL, controlling the inflammatory response rather than solely restraining parasite replication.

## 3. Nanostructures as carriers for anti-leishmanial drugs

Nanomedicine offers the possibility to enhance drug efficacy while decreasing their toxic effects. They are currently being used in anti-cancer chemotherapy due to their profile of security and good tolerance, for instance Nab-paclitaxel (Abraxane) as a first-line treatment of metastatic pancreatic carcinomas and in second-line therapy for metastatic breast cancer. Nano-based drug delivery optimization for tuberculosis treatment has also been thoroughly studied [51]. The application of this strategy to treatment of CL can further be extended to tissue regeneration possibilities. This section gives a critical analysis of nano-based strategies which have been assessed for CL treatment.

**Figure 6.**
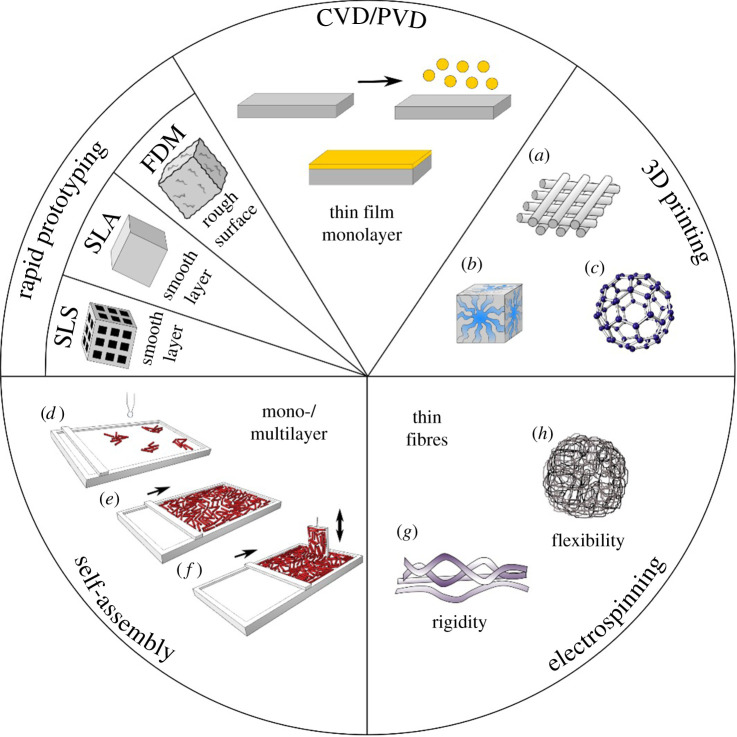
Surface modification techniques. These techniques include rapid prototyping (RP) methods, such as selective laser sintering (SLS), selective laser ablation (SLA), fused deposition modelling (FDM), chemical and physical vapour deposition (CVD, PVD), 3D printing methods resulting in tailor-made layered (*a*), cubic (*b*) and spherical (*c*) structures; various self-assembly methods, that is, Langmuir–Blodgett technique for monolayer formation including spreading of polymer solution (*d*), compression to single monolayer (*e*), and film transformation onto substrates (*f*); and electrospinning of rigid (*g*) and flexible (*h*) polymers. Copyright Elsevier 2022 [52].

Although manufacturing of nanomaterials is not addressed in detail, the most prominent methods are shown in figure 6. Today, 3D printing, electrospinning and rapid prototyping are used. Nanostructured films are available via chemical and physical vapour deposition or various self-assembly methods (such as Langmuir–Blodgett) [52]. In addition, lithography, particularly photolithographic techniques have been developed to generate tailored micro- and nanostructures [53].

In the following sections, nanoparticles, nano/microfibres and hydrogels produced for Leishmaniasis treatment will be discussed in more detail.

### 3.1. Nanoparticles

Poor performance of drugs poses a major challenge for delivery of drugs to specific cells. The biological barriers encountered by the therapeutic modalities favour dissemination of the disease like intramacrophage location of parasite, lack of oral bioavailability, permeability across the cutaneous tissue and active efflux of the drug. Indeed, hydrophilic drugs such as paromomycin cannot pass the skin in its free form and are thus unable to kill parasites within macrophages [54]. Moreover, adverse side effects of current anti-leishmanial drugs led to increased interest in the use of nanomedicine for leishmaniasis therapy. Nanomedicine may be administered through oral, IV or through cutaneous routes with IV being the most frequent route.

Nanoparticles (NPs) offer the possibility of overcoming physiological barriers to enter cells, improving drug solubility, multiple drug loading with high drug content, enhanced stability, control drug distribution in the body, as well as good circulation throughout the body. In CL, the parasite is located in skin macrophages, and dendritic cells, including Langerhans cells, together with lymph nodes and mucosal cells in mucosal CL. Therefore, the effectiveness of drugs may be increased by specifically targeting these tissues. Indeed, control over the chemical and structural properties of NPs allow the specific delivery of multiple drugs to targeted sites via surface modification with biomolecules and therapeutic drugs [55]. To reduce adsorption of blood proteins and to decrease unspecific biodistribution, NPs may be functionalized with biocompatible materials. The size and surface charge of NPs may be controlled for more specific accumulation and biodistribution. For instance, NPs with size smaller and larger than 150 nm are retained in the liver and the spleen, respectively [56]. Positively charged NPs are trapped in liver, spleen and lungs [57]. In addition, the action of drugs may be controlled through modification of NPs with ligands such as antibodies, peptides, lipids, carbohydrates and nucleic acid which allow recognition of pathophysiological markers of leishmaniasis on the parasites or infected cells [58].

The use of NPs can increase the effectiveness of leishmaniasis treatment, reduce toxic side effects and frequency of the medications [59]. Chitosan is an interesting material for NPs fabrication due to its positive charge which favours adsorption by negatively charged cell membranes [60]. In addition, it showed intrinsic anti-leishmanial activities against *Leishmania* parasites [61]. *In vivo* studies in *L. major* murine model confirmed that chitin was a better immunomodulator compared with chitosan due to enhancement of IL-10 and TNF-α productions by chitin microparticles (MPs) compared with chitosan MPs [62]. Metal NPs especially Au and Ag have high anti-leishmanial activities due to the metal-oxidation capability, causing greater damage to cell membranes [55]. Table 2 summarizes the main NPs drug delivery systems investigated for leishmaniasis treatment.

**Table 2.** Summary of main NPs drug delivery systems studied for leishmaniasis treatment.

NP system (average particle size)	*Leishmania* species	drug-loaded (% encapsulation efficiency)	target (tested *in vitro*)	main findings
metal NPs				
gold NPs (30 nm) [63]	*L. tropica*	—	promastigotes	significant anti-leishmanial activity with maximum of 75% growth inhibition
silver NPs(35 nm) [64]	*L. donovani*	Miltefosine		resulted in loss of structural integrity in treated promastigotes
				IC_50_: 12.5 µM MILT + 50 µM AgNP promastigote
silver NPs + UV [65]	*L. major*	—		highest pronounced inhibitory effect using combinatorial therapy versus Ag NPs only
selenium NPs [66]	*L. major*	—	promastigotes and amastigotes	1.62 ± 0.6 µg ml^−1^ promastigote
				4.4 ± 0.6 µg ml^−1^ amastigote
				limit localized cutaneous lesions.
inorganic NPs				
MgO NPs(50 nm) [67]	*L. major*	—	promastigotes	decrease promastigote cell viability
				compared with MgO NPS, glucose-coated MgO NPs reduced the expression of Cpb and GP63 genes more significantly
ZnO NPs (20 nm) [68]		—		induced apoptosis in a dose and time-dependent manner
				37.8 µg ml^−1^ promastigotes
TiO_2_-NPs (170 nm) [69]		Glucantime^®^	promastigotes and amastigotes	13-fold and fourfold decrease in promastigote and amastigote proliferation, respectively
TiO_2_-Ag NPs [70]	*L. tropica*	meglumine antimoniate (MA)		decreased proliferation of promastigotes by two- to fivefold in contrast to use of MA alone
bovine serum albumin NPs (180 nm) [71]	*L. amazonensis*	amphotericin B (95%)		higher effectiveness against amastigotes than promastigotes
				no tissue toxicity compared with the use of free drug
liposomes				
liposomes [72,73]	*L. major*	paromomycin (60%)	promastigotes and amastigotes	65.32 µg ml^−1^ promastigotes
				24.64 µg ml^−1^ amastigotes
				complete healing with significantly lower parasite load in spleen
		meglumine antimonial (25–38%) average size: 150 nm		10.5 µM amastigotes
				>9000 µM promastigotes
				higher selectivity index
				concentration required to kill 100% of the intracellular amastigotes was ≥40-fold lower with MA encapsulated liposomes compared with the free drug
				significantly increased uptake in infected macrophages
solid lipid NPs				
solid lipid NPs (299 nm) [74]	*L. major* and *L. tropica*	paromomycin (42–46%)	promastigotes	1600 µg ml^−1^ for *L. major* and *L. tropica* promastigotes
polymeric NPs				
PLGA (365 nm) [75]	*L. amazonensis*	amphotericin B	promastigotes *in vivo*	significant reduction in the number of parasites on the paws of rats
				increased cell viability and reduced the number of infective cells compared with free drug
				similar efficacy as from AMB in reducing paw diameter of rats
chitosan	*L. amazonensis* [76]	amphotericin B	*in vivo*	significant reductions in the lesion size and in the parasite burden in all evaluated organs
				effective in diminishing the toxicity of AMB
	*L. major* [77]	amphotericin B (90%) Average size: 112 nm	promastigote and amastigotes *in vivo*	reduction of cellular toxicity by 100%
				perfect wound healing
				improvement of *L. major* infected macrophage by 81%
	*L. major* [78]	paramomycin (15–84%)	promastigotes and amastigotes	reduced parasite burden
		average size: 246–600 nm		high selectivity indices, i.e. simultaneous favourable safety toward macrophages and vigorous toxicity against *L. major* amastigotes
chitosan-coated NLCs (103.7–143 nm) [79]	*L. donovani*	ursolic acid (UA) (88%)	amastigotes	parasite burden suppression by 98.75%
chitosan NPs (287–295 nm) [80]	*L. tropica*	meglumine antimoniate (58–63%)		IC_50_ of the mannose-targeted nanoparticles 14.41-fold lower than the glucantime
				macrophage uptake was 33.7-fold higher with the mannose-targeted nanoparticles as compared with the glucantime
				significantly improved biocompatibility of drug in nanoparticles as compared with glucantime

There are a few promising NPs containing the biopolymer lignin that may play a crucial role in the future. In the wake of global climate and resource pressures, it is more urgent than ever to also design new research approaches as sustainably as possible. Lignin is the second most abundant and renewable natural biopolymer [81]. One quarter of wood contains lignin along with cellulose-related components in trees and plants [82]. Current applications cover bio-based chemicals and high-performance polymer nanocomposites [83]. Promising lignin-based composites are under investigation for drug delivery and biomedical applications [84]. For this purpose, lignins can be converted into lignin-based hydrogels and/or nanoparticles (LNP) which are increasingly being studied for controlled drug release [85] for different types of molecules. The most promising lignin-based approaches are presented in table 3.

**Table 3.** Summary of LNPs drug delivery systems used for disease treatment.

LNP system (preparation method, lignin origin/isolation process and average size)	drug-loaded (% encapsulation efficiency)	loading capacity (%)	main findings
kraft LNPs dialysis technique (129.88–203.5 nm) [86]	irinotecan (67.6 ± 2.0)	13.6 ± 0.6	LNPs reduced the IC_50_ value of irinotecan by almost threefold
organosolv-type LNPs (stabilized by citric acid) self-assembly method (85.9–104 nm) [87]	curcumin (92 ± 4)	—	*in vitro* release experiments showed that curcumin-loaded LNPs achieved high stability in simulated gastric fluid
enzymatically hydrolysable lignin (EHL) hollow NPs dialysis technique (396–405 nm) [88]	doxorubicin-hydrochloride (>60)	>12.5	encapsulation of the drug was enhanced by the pore volume and surface area
LignoBoost^TM^ softwood kraft LNPs dialysis technique (221 ± 10 nm) [89]	Sorafenib (68 ± 19)	7 ± 2	morphology of the drug-loaded pLNPs did not change compared with empty LNPs. Less than 4% of the pure drug was released at pH 5.5 and 7.4, due to low solubility of SFN in aqueous solutions
	benzazulene (77 ± 10)	8 ± 1	anti-proliferative effect of benzazulene in different cell lines (EA.hy926, MDA-MB-231, MCF-7, PC3-MM2 and CaCo-2) after incorporation into LNPs was enhanced
alkali LNPs (131.2–183.6 nm) self-assembly method [90]	resveratrol (>90)	23.8	addition of Fe_3_O_4_ within the NPs increases the stability, accumulation and anti-cancer effect of resveratrol significantly improved compared with free agents

**Figure 7.**
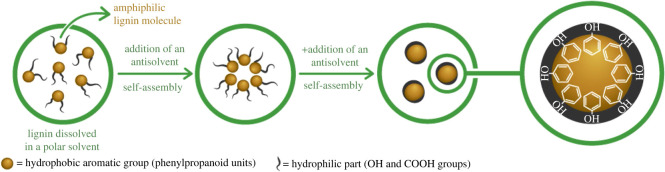
A schematic proposal for the formation of LNPs [91]. Copyright Elsevier 2022.

The most common method used for LNP belongs to the group of self-assembly techniques. For this, the lignin is dissolved in an organic solvent, and the LNP are subsequently formed by adding an anti-isolating agent, as shown in figure 7 [91].

The formation of micelles can also be achieved by dialysis process as recently shown by Gericke *et al*. For this purpose, organosolv lignin was embedded in a biocompatible polysaccharide matrix (xylanphenyl carbonate and cellulose acetate phthalate) to form hybrid LNP, promising structures for pharmaceutical applications [92].

### 3.2. Micro/nanofibres

Compared with NP delivery systems, micro- and nanofibres have not been extensively investigated for anti-leishmanial drug loading. Micro- and nanofibrous mats have high surface area allowing high drug encapsulation efficiency and may be compressed to form oral tablets, thereby increasing patient compliance and reducing costs linked to conventional chemotherapy which requires hospitalization [93].

Laha *et al.* [93] successfully fabricated compressed cross-linked amphotericin B-loaded gelatin nanofibrous oral tablets which exhibited excellent zero order drug release kinetic for up to 10 days. To prevent initial burst release, the tablet was coated with sodium alginate (SA) to control initial fluid penetration [94]. SA coating also increased the stability of the tablet under physiological pH.

The topical application of drugs for CL requires drug penetration within the dermis, where the parasites are internalized into macrophages. Using a polyvinyl alcohol (PVA)-based electrospun mat as chalcone delivery system [95], it was found that the nanosystem allowed mostly retention of the drug in the upper skin layer while the nanoemulsion form penetrated much deeper. *In vitro* testing using amastigotes of *L. amazonensis* revealed greater efficiency of parasitic growth inhibition compared with the free forms of the chalcones.

Alishahi *et al*. [96] engineered a topical anti-leishmaniasis drug delivery system to treat CL using electrospun core–shell nanofibres made up of the biocompatible polymers polyethylene oxide (5% w/v), gelatin (1% w/v), polyvinyl alcohol (PVA, 6% w/v) and chitosan (3% w/v). A maximum of 20% (w/w) glucantime was loaded and 84% of the drug was released *in vitro* within the first 9 h. The amount of glucantime loaded into the fibres did not alter their cytotoxicity towards NIH3T3 cell line. The topical delivery mats were non-toxic to fibroblast cells (NIH3T3) and were able to eliminate 78% of *L. major* promastigotes *in vitro*.

Core–shell nanofibres made up of polylactic acid (PLA) and polyethylene glycol (PEG), loaded with amphotericin B were developed by Gonçalves *et al*. [97]. It was synthesized by solution blow spinning (SBS) and expressed high *in vitro* anti-leishmaniasis activity against *L. amazonensis* and *L. braziliensi*s. It was reported that the nanofibres loaded with 1, 0.5 and 0.25% of amphotericin B (Amp B), respectively, killed all promastigotes in the culture media. *In vitro* controlled release of Amp B showed some promising characteristics. Release of the antifungal and anti-leishmaniasis drug started within 1 h, followed by a gradual increase in rate till day 7.

Liang *et al*. [98] reported on the electrospinning of poly(ε-caprolactone) (PCL)-grafted lignin (PCL-g-lignin) copolymer CNFs which showed excellent antioxidant and anti-inflammatory properties and low cytotoxicity. PCL-g-lignin CNFs inhibited the formation of reactive oxygen species and activated antioxidant enzyme activity through an autophagic mechanism. The nanofibrous PCL-lignin membrane can be implanted during arthroscopic surgery and provided effective osteoarthritis therapy. Arginine-derived lignin NF was prepared by electrospun lignin NF, which has suitable viscosity that can be used for enhanced spreadability of topical application. *In vivo* wound healing test was demonstrated in rats. The arginine-based lignin NF accelerated wound healing and increased re-epithelialization, collagen deposition and angiogenesis compared with lignin NFs and arginine [99]. Ago *et al*. [100] developed a fibre material composed of lignin, polyvinyl alcohol and cellulose nanocrystals (CNC). They investigated the morphology of the electrospun lignin/PVA/CNC fibres, focusing on understanding the distribution of polymers on the fibre surfaces as a function of fibre composition. The fibre properties of this composite material and its fibre surface characteristics, such as surface energy, make it an extremely interesting candidate for the development of a fibre material for controlled drug delivery. Electrospun softwood kraft lignin/polyamidoamine dendrite (PAMAM) polymer blends exhibited strong interaction between the phenolic groups of lignin with the amino groups of PAMAM [101]. This not only improved the mechanical and thermal properties of the mats, but also enhanced adhesion to the profile required for a drug release material. A highly stretchable electrospun lignin-based biomaterial was developed containing poly(methyl methacrylate) (PMMA) and poly(ε-caprolactone) (PCL) which showed high biocompatibility with human skin fibroblasts [102]. A lignin copolymer synthesized using β-butyrolactone and/or ε-caprolactone was further coupled with poly(3-hydroxybutyrate) (PHB) to prepare PHB/lignin NFs using electrospinning techniques [103]. The obtained PHB/lignin NFs showed enhanced tensile strength and elongation with good biodegradability and biocompatibility.

#### 3.2.1. Electrospun wound dressing

Rahimi *et al*. [104] designed an electrospun wound dressing made of chitosan (CS)-polyethylene oxide (PEO) nanofibres loaded with berberine and assessed its anti-leishmanial activity against *L. major in vitro*. The nanofibres were found to be non-toxic, biocompatible and did not hamper the proliferation of fibroblast cells. In addition, the nano-scaffolds had prolonged drug release capacity up to 14 days with 50% release within the first 18 h and 80% at day 3. It was observed that a load of 20% berberine (w/v) in the nanofibres significantly inhibited growth of promastigotes *in vitro* (IC_50_ = 0.24 µg ml^−1^). Tabaei *et al*. [105] also worked on CS-PEO-berberine nanofibres and demonstrated accelerated wound healing of *Leishmania* ulcers in murine model. Efficiency of the wound dressings, also known as nano-bandages, was assessed in BALB/c mice infected with *L. major*. Similar to previous findings [104], the nanofibre mat was biocompatible and demonstrated significant berberine release rate (from 70.75% on the first day to 80.8% on the third day) up to two weeks. The nano-bandage proved its efficacy by reducing skin ulcer through promotion of wound healing and reduced parasitic load in the ulcers. Considering the integration of antimicrobial active electrospun fibres like for instance, Mg(OH)_2_-NS PEO/PCL (polycaprolactone), that is reported to be efficiently acting also against *S. aureus* and *E. coli* [106] and the combination with a proangiogenic hydrogel and wound healing monitoring microenvironment sensor like previously reported [107], there is high potential to further complete the functionality of a CL-optimized wound healing bandage.

### 3.3. Hydrogels

Hydrogels were previously reported as suitable carriers for topical drug delivery [108]. Patients usually seek medical attention after developing well-established lesions which are highly inflamed, ulcerated in some cases, with lots of damaged tissues and a high parasitic load. Compared with NP formulations which are often administered intravenously, hydrogel formulations are more easily administered by directly placing the latter onto the skin lesions. Hydrogels have shown good biocompatibility and high water content that mimic the features and properties of body tissues (due to their ability to swell and hydrophilic nature) and highly resemble the wound extracellular matrix.

#### 3.3.1. Enhanced ease of administration and lower cost via topical formulations

The three-dimensional microporous structure of a hydrogel matrix allows for drug encapsulation. This provides protection from hostile environment to preserve the drug or compound's full potential, and in some cases, enhance their anti-leishmanial activity. Poly(*N*-2-vinyl-pyrrolidone) (PVP) and poly(vinyl alcohol) (PVA) based clay-hydrogels have been synthesized to encapsulate an antimoniate drug, *N*-methyl glucamine using gamma irradiation [109]. PVP : PVA hydrogel (50 : 50) showed a higher and steady release of *N*-methyl glucamine even after 15 h in the presence of 1.5% clay and reduced the leishmaniasis lesion by 99% in a murine model previously infected with *L. amazonensis* amastigotes. Similarly, cobalt-60 gamma irradiation was used to cross-link as well as sterilize PVP, polyethylene glycol (PEG 400), agar and laponite RD clay loaded with amphotericin B (Amp B). The latter was found to be released in a sustained manner over 12 h and maintained its structure and activity after exposure to irradiation and elevated temperature. A load of 25.1 nM of Amp B in 1.324 g l^−1^ hydrogel exerted the best anti-leishmanial activity against *L. amazonensis* promastigotes with a 100% growth inhibition within 48 h [110]. Amp B has also been loaded within PVA hydrogels with good water permeability (452 ± 10 g m^−2^ d^−1^) which is an essential feature for the development of an effective wound dressing to retain a moist environment for better healing and absorption of any excessive exudates. In addition, the hydrogels showed high ability to potentially hamper secondary bacterial or fungal infections by acting as barrier against microorganisms. A rather slow and gradual release of Amp B (74% after 97 h) was reported but did not impact on the system's anti-leishmanial activity. They were found to be highly toxic against *L. amazonensis* and *L. braziliensis* with a promastigote death rate of 100% and 99%, respectively, within the first 24 h [111].

Lalatsa *et al*. [112] developed an anti-leishmanial self-nanoemulsifying drug delivery system (SNEDDS) hydrogel. The nano-enabled hydrogels were designed to release antiprotozoal buparvaquone (BPQ) topically. The hydrogel system improved the solubility of hydrophobic BPQ. The 1% BPQ-SNEDDS gels were applied on lesions of infected BALB/c mice for 7 consecutive days and were shown to be highly effective against *L. amazonensis*, with a 99.989 ± 0.019% decrease in parasite load. The BPQ-SNEDDS hydrogels did not trigger any inflammation and showed good healing capabilities.

Recently, Risedronate monosodium monohydrate (Ris)-hydroxypropyl methylcellulose (HPMC) and Eudragit EPO(EuE)-Ris-HPMC hydrogels were assessed to serve as potential new curative treatment against CL. Decrease in leishmanial lesion size and lower level of *L. amazonesis* amastigotes were observed in mice treated with Ris (20 mg ml^−1^)-HPMC (2%) and EuE (40 mg ml^−1^)-Ris (20 mg ml^−1^)-HPMC (2%). The parasite load at the end of the treatments was higher in the control group compared with the two systems tested (parasite suppression rate of 69.5% for Ris-HPMC and 73.7% for EuE-Ris-HPMC). Histological analysis showed vacuole formation in parasites treated with EuE-Ris-HPMC, suggesting autophagy as the mechanism responsible for amastigote death [113].

#### 3.3.2. Increased skin permeability

To increase skin permeability of amphotericin B, Zare *et al*. [114] developed a dissolvable microneedle patch made of PVP and carboxymethyl cellulose. The microneedles on the patch were able to penetrate rat skin at a depth of 303 ± 8 µm and then dissolved, releasing the encapsulated amphotericin B. The micropores created by the microneedles in the rat skin did not cause significant cell damage and were rapidly resealed within 30 min. The cytotoxicity of the patch was also assessed on HT-29 cells and was found to have no negative effects. The microneedle system demonstrated effective transdermal delivery with an anti-leishmanial activity up to 86% parasite death.

#### 3.3.3. Infection management and biofilm elimination

Tavakolian *et al*. [115] developed highly absorbent antibacterial and biofilm-disrupting carboxyl-modified cellulosic hydrogels for wound healing applications. The hydrogels were surface modified with polylysine and showed antibacterial properties against both *Staphylococcus aureus* and *Pseudomonas aeruginosa* and were able to kill approximately 99% of the bacteria after 3 h of exposure. In addition, the hydrogels showed good absorption capacity and promoted the proliferation of fibroblasts. In another study, novel antimicrobial hydrogels composed of bacterial cellulose and poly(3-hydroxy-acetylthioalkanoate-co-3-hydroxyalkanoate) (PHACOS) were developed [116]. The latter displayed fibroblast cell viability of over 85%, had elastic properties comparable to the skin, and optimum swelling properties for absorbing wound exudates. Double-network adhesive hydrogels based on cellulose and 3,4-dihydroxyphenylalanine (DOPA)-cation copolymer were successfully fabricated with tissue-like Young's modulus below 20 kPa [117]. The catechol-cation cooperation effect enhanced the wet adhesion property of the hydrogels to skin. The hydrogels displayed rapid haemostasis, good biocompatibility and antibacterial activity for promising wound healing applications.

Lignin-derived hydrogels have been synthesized by a variety of methods, such as hydrothermal methods, ultrasonic polymerization, wet spinning, ultrasonic and various cross-linking methods, such as esterification reaction, copolymerization with other polymers such as acrylic acid [118–120]. A study by Mahata *et al*. [121] confirmed that copolymerization with a triazole component improved the antibacterial and antibiofilm activity of lignin, resulting in down-regulation of interleukins, especially IL-1, in lipopolysaccharide (LPS)-induced macrophage cells and reduction of inducible nitric oxide synthase (iNOS) levels. The study was also supported by Western blotting and NF-KB analyses. This novel lignin-based hydrogel has been shown *in vivo* to be able to prevent burn wound infections, promote healing and serve as an anti-inflammatory dressing material. In addition, lignin from two different sources was cross-linked by different methods, attempting to form bulk and membrane hydrogels [122]. Results confirmed appropriate water vapour permeability, antioxidant activity and antimicrobial activity of the membranes indicating their potential use as wound dressing materials. To create an effective antimicrobial agent in the form of a dressing for the treatment of chronic wounds, Zmejkoski *et al*. [118] developed a composite hydrogel of bacterial cellulose and dehydrating polymer of coniferyl alcohol, a monomer of lignin. The novel composite showed inhibitory or bactericidal effects against selected pathogenic bacteria, including clinically isolated bacteria. The highest rate of release of dehydrating polymer of coniferyl alcohol was in the first hour, while after 24 h there was still a slow release of small amounts of dehydrating polymer of coniferyl alcohol from composite hydrogel of bacterial cellulose and dehydrating polymer of coniferyl alcohol during 72 h monitoring. All results confirmed that the composite is a promising hydrogel for wound healing.

## 4. Multiple nano-drug delivery systems for simultaneous *Leishmania* treatment and wound repair

**Figure 8.**
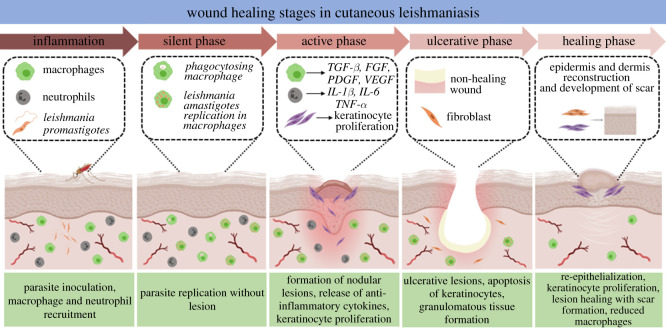
Scheme summarizing lesion development in CL.

CL causes important skin damage with scarring. Lesions start with a small erythema which progresses to a papule, followed by a nodule, an ulcerative/non-ulcerative lesion dependent on the species of the parasite [1]. Lesion development consists of five main stages namely the initial inflammatory phase, silent phase, active phase, ulcerative phase and healing phase (figure 8).

In the initial inflammatory phase which may last between a few weeks to months, no pathological change is noted in the skin [123] and macrophages migrate to the bite site and act as host cells for the *Leishmania* amastigotes [124]. The silent phase is characterized by parasite proliferation without formation of lesions. In the active phase, a nodular lesion is formed accompanied by a decrease in parasite load. At this stage, other inflammatory cells such as T cells, DCs [125] and Langerhans cells also infiltrate in the dermis. The final stage marks the development of an ulcerative lesion covered by apoptosed keratinocytes, dried exudate and a mixture of live and dead amastigotes. The dermis is transformed to dermal granulomas [126].

Immunological responses play crucial roles during the wound healing process in CL. Cytokines IL-10 and TGF-β have been reported to play paradoxical roles in immunity against CL and in promoting wound healing [127]. Indeed, IL-10 and TGF-β are responsible for parasite persistence in CL through suppression of immune responses in CL. On the other hand, they are important accelerators of the physiological wound healing cascade. As a result, there has to be a compromise in IL-10 and TGF-β concentrations to enhance parasite elimination along with accelerated wound healing.

### 4.1. Need for multiple drug platform for anti-leishmanial activity

#### 4.1.1. Lower toxicity and improved efficiency

Nano-drug delivery systems have been loaded with multiple drugs for enhanced efficiency and better therapeutic effects. Tripathi *et al.* [128] loaded both amphotericin B and miltefosine into chitosan-coated lipid nanocarriers. Chitosan coating slowed down the release of the drugs and also increased uptake in cells compared with the free drug. Electrostatic interaction with macrophages and combinatorial effect of dual drugs led to improvement *in vivo* parasite inhibition of 85 ± 4.20% versus 53.26 ± 2.5% for the free amphotericin B. Due to location of amphotericin B within the nanocarriers, the drug-loaded NPs showed insignificant haemolysis compared with the free drug.

Similarly, Parvez *et al*. [129] developed an oral drug delivery system made up of chitosan-grafted solid lipid nanoparticles (Cs-SLN) loaded with amphotericin B and paromomycin, two anti-leishmanial drugs (entrapment efficiency 95.20 ± 3.19% and 89.45 ± 6.86% respectively). Cs-SLN was reported to be less cytotoxic than free amphotericin B, stable in gastrointestinal tract fluids and the chitosan coating enhanced muco-adhesion of the nanoparticles. An initial burst release of 27.6% amphotericin B and 34.4% paromomycin was observed within the first 6 h followed by a constant and slow drug release up to 3 days. Cs-SLNs proved its efficacy with a higher anti-leishmaniasis activity (IC_50_ 0.018422 ± 0.005928 µg ml^−1^) compared with free amphotericin B (IC_50_ 0.316039 ± 0.026423 µg ml^−1^) and highly inhibited growth and reduced *L. donovani* intracellular amastigotes load within macrophages (92.35%).

#### 4.1.2. Targeting secondary bacterial infections

Ulcerative wounds among CL patients become infected with bacterial and fungal infections from the environment often when the wounds are not kept hygienically. These secondary infections are responsible for additional pain, secretion, pruritus and burning sensation. It may delay wound healing and complicates diagnosis of CL [130].

It has been reported that the normal flora of the skin can influence allergic and autoimmune responses, assist wound healing and initiate antimicrobial defence. The skin microbiota in CL patients develop dysbiotic skin microbiota which essentially reduce the diversity of microbial species and allow the predominance of *Staphylococcus* and or *Streptococcus* [131]. The dysbiotic skin microbiota heighten skin inflammatory responses. When a wound becomes infected, and not properly treated, it takes significantly longer time to heal [132,133].

In a recent study in Ghana [134], 42 secondary bacteria from 48 CL patients with open wounds were isolated. *Staphylococcus aureus* was the most predominant among all the bacteria isolates. However, several pathogenic bacteria species were also detected, such as *Bacillus subtilis*, *Klebsiella pneumoniea, Enterobacter cloacae*, *Aeromonas* spp *Serratia liquefacien*, *Providencia rettgeri* and *Cronobacter* spp. It was intriguing to observe that the majority of these bacteria isolates were resistant to beta-lactam antibiotics and the third-generation cephalosporin. Notably, 84.6% of the *S. aureus* isolates were methicillin and ciprofloxacin resistant while 92.3% were resistant to ampicillin. Among a cohort of 25 CL patients, the presence of *Staphylococcus aureus*, *Pseudomonas aeruginosa*, *Enterococcus faecalis*, *Streptococcus pyogenes* and *Candida parapsilosis* was detected. [130]. The presence of bacteria in the ulcer border and ‘pain’ and ‘pruritus’ had no influence on wound closure, the presence of ‘secretion’ and ‘burning sensation’ delayed epithelialization time but not total healing time. Kariyawasam *et al*. [135] observed that in 61% of CL patients, there was the co-colonization fungal genera such as *Malassezia, Aspergillus*, *Candida* and *Cladosporium*. In about 50% of the patients, the fungal infections were responsible for inflammation, and fungal–bacterial infections complicated the diagnosis of CL.

An effective remedy should be able to efficiently tackle the parasite load and treat any secondary infections occurring simultaneously, thus reducing the drug and financial burden on carers and patients.

Different prototype drug delivery systems have been assessed as potential alternative treatment that could both eradicate the *Leishmania* parasites and other microbial pathogen present in the lesion area. Curcumin-loaded self-emulsifying drug delivery system (cu-SEDDS) formulations have demonstrated both anti-leishmanial activity against *L. tropica* (IC_50_ ranging from 0.19 to 0.37 depending on concentration of curcumin-loaded) and antibacterial potential against Gram-positive (*Staphylococcus aureus*) and Gram-negative (*Escherichia coli*, *Pseudomonas aeruginosa* and *Klebsiella pneumoniae*) pathogens. The amplified antiparasitic and antimicrobial properties of cu-SEDDS, compared with free curcumin, were attributed to the improved solubility of hydrophobic curcumin which enhanced cellular intake by parasites and microbes [136].

To overcome barriers of parasite resistance to treatment, unwanted side effects from current leishmaniasis medications and microbial wound infections, Costa *et al*. [137] formulated non-cytotoxic biodegradable polybutylcyanoacrylate nanoparticles coated with polymyxin B (PBCAnp-polB) which expressed both anti-leishmanial and antimicrobial activity. Inhibition of *L. amazonensis* promastigotes were promoted by the PBCA nanoparticles. Moreover, the polymyxin B bound to the nanoparticles stayed active and was responsible for the antibacterial activity against *E. coli, P. aeruginosa* and *K. pneumoniae* [137]. Titanium dioxide (TiO_2_) and silver oxide (Ag_2_O) nanoparticles were also reported to have anti-leishmanial activity with simultaneous high microbial growth inhibition properties [138]. Ag_2_O nanoparticles (Ag_2_Onp) are said to enhance activity of common antibiotics such as penicillin G, vancomycin, amoxicillin and erythromycin and inhibit growth of multi-drug-resistant bacteria such as methicillin-resistant *Staphylococcus aureus* (MRSA) [139]. When Ag_2_Onps enter a bacterium, they interact with the sulfur and phosphorus groups within DNA molecules and make them lose their replication properties. Consequently, DNA damages occur as the bacterial cell cycle is stopped at the G2/M phase [140]. Finally, apoptosis is induced due to inhibition of ATP synthesis and the presence of reactive oxygen species (ROS) causing more damage to DNA and RNA molecules, lipid peroxidation and amino acid oxidation [141]. ROS also occur at the surface of TiO_2_ nanoparticles (TiO_2_np) causing lipid peroxidation strongly affecting bacterial cells. Another mechanism by which TiO_2_nps eradicate bacteria is through the photocatalytic reactions occurring at their surface which increase cell membrane permeability. Essential components thus leak out of the bacterial cell causing death [142,143].

Antimicrobial photodynamic therapy (aPDT) that follows a similar ROS-based mechanism via photoactivation of photosensitive molecules such as porphyrins, phthalocyanines and hydrophilic benzophenoxazine analogues, have shown efficacy in the eradication of *Leishmania* and Gram-positive and Gram-negative bacteria [144–146]. But this approach requires specialized equipment and skilled personnel or trained patients that need to expose the illuminating LED source to the defined region following a precise standard protocol.

### 4.2. Wound healing in leishmaniasis

A number of small natural molecules have been repurposed to target simultaneously healing of leishmanial wounds and apoptosis of *Leishmania* parasites. Recently, secondary metabolites such as lignans, alkaloids, phenolic derivatives (chalcones and flavonoids), and terpenes (iridoids, sesquiterpenes, diterpenes, triterpenoids and saponins) have been reported to possess anti-leishmanicidal activity [147–151]. Interestingly, flavonoids and alkaloids display dual anti-leishmanial and wound healing properties.

#### 4.2.1. Inflammatory phase

##### 4.2.1.1. Flavonoids

Curcumin, which has long been used to accelerate wound healing by enhancing fibroblast proliferation, granulation tissue formation, collagen deposition and tissue remodelling [152], was recently shown to be cytotoxic to several strains of *Leishmania* including *L. major, L. tropica* and *L. infantum* by inducing cell cycle arrest at G2/M phase [153]. In fact, curcumin caused the formation of reactive oxygen species (ROS) and increased the concentration of cystolic calcium which led to DNA fragmentation of the parasites [154]. Chaubey *et al*. [155] successfully used curcumin-loaded chitosan NPs to suppress parasite replication *in vivo* to a greater extent as a result of higher macrophage uptake compared with free curcumin.

##### 4.2.1.2. Sesquiterpenes

Artemisinin and its derivatives artemether and artesunate showed *in vivo* anti-leishmanial activity against *L. amazonensis* parasites [156] and limited *in vitro* amastigote and promastigote activity [157]. The mechanism of action is via production of free radicals which induces parasite death in the presence of iron sources. Artemisinin has been loaded in nanofibrous mats and successfully used to accelerate *in vivo* wound healing through its anti-inflammatory and antibacterial properties [158]. Another sesquiterpene which inhibited *L. amazonensis* parasite growth is parthenolide [159]. The latter has been found to display a variety of anti-inflammatory and immunomodulatory effects [160]. *In vitro* studies revealed that parthenolide inhibits the NFkB pathway by targeting the inhibitor (I)kB kinase activation or IkBa degradation [161].

##### 4.2.1.3. Triterpenoids

Glycyrrhizic acid extracted from licorice effectively reduced parasite burden in infected macrophages through inhibition of Cox-2, leading to decreased prostaglandin E2 biosynthesis, which in turn resulted in increased NO generation in the infected macrophages, thus arresting parasite survival [162]. It also has anti-inflammatory properties through inhibition of the expression levels of pro-inflammatory cytokines (TNF-α, IL-1β and IL-6). It also regulated cell proliferation through its influence on ERK1/2 signalling pathway [163].

#### 4.2.2. Proliferative phase

##### 4.2.2.1. Alkaloids

Berberine, found in a number of plants such as Annonaceae, Berberidaceae and Menispermaceae, is one of the alkaloids displaying the highest anti-leishmanial activity [164]. It has been shown to effectively eliminate *L. major* parasites in macrophages at a concentration of 10 µg ml^−1^ and was also effective against lesions caused by *L. panamensis* in rats [165]. In addition, the alkaloid has antibacterial properties and can inhibit Gram-positive and Gram-negative bacteria [166]. Recently, Zhang *et al.* [167] showed that berberine reduced inflammation by inhibiting the expression of NF-κB, TNF-a and IL-6, but increased the expression of VEGF and CD31, which enhanced proliferation of vascular endothelial cells, and also increased SMA, which promoted proliferation and migration of fibroblasts.

##### 4.2.2.2. Polyphenols

Resveratrol, primarily found in grapes, showed anti-leishmanial activity against promastigotes *in vitro* and was effective against intracellular amastigotes [168]. A derivative tested by Antinarelli *et al.* was found to be more active than miltefosine (IC_50_ < 3.0 µg ml^−1^). The compound acted through mitochondrial potential depolarization, plasma membrane permeabilization, interference in the progression of the cell cycle and accumulation of autophagic vacuoles [169]. In several wound healing studies, it was shown that resveratrol promoted granulation tissue formation and had strong angiogenic properties [170]. Moreover, it reduced oxidative stress and promoted fibroblast proliferation and migration [171]. It decreased scar formation in a rat skin model by suppressing inflammation and led to well-organized collagen deposition [172].

## 5. Future perspectives: drug delivery in combination with wound healing monitoring

### 5.1. Smart hydrogels to detect and treat bacterial infections and monitor CL wound status

Secondary bacterial infection is one common complication with Leishmaniasis and should be detected as early as possible allowing specific treatment in time to avoid a chronic course of the disease, further scarring [173] and misuse/overuse of broad-spectra antibiotics.

At the moment, it is not feasible to ensure a proper monitoring of secondary bacterial infection for patients in remote and rural areas, because they are costly and require a wound swab sampling by trained medical personnel unavailable in limited healthcare settings. Standard microbiological detection requires time-consuming procedures like selective cultivation methods often combined with chromogenic differential culture medium [174] or faster advanced molecular diagnostic methods mainly based on polymerase chain reaction (PCR) [175]. The delay generated by this method may result in missing the optimal treatment window that would offer the best chance to eradicate pathogenic bacteria before they build up robust biofilms in leishmaniasis ulcera which leads to chronic bacterial infections.

Efficient monitoring of Leishmaniasis wounds regarding such bacterial infections should be autonomous and low-cost point-of-care systems. The targets of such sensors to identify bacterial infection are ranging from the rather unspecific temperature and pH shift [176,177] to specific bacterial enzymes produced by unique bacteria to be detected [178].

Bacterial enzymes have been addressed by several enzyme responsive smart biomaterials developed in the past including enzyme responsive polymersomes [179] and colorimetric enzyme substrate reporter units coupled to a chitosan film [180,181]. This enzyme responsive hydrogel approach was expanded to a multiplex approach that enables the simultaneous differentiation of various bacterial enzymes and bacteria via coupling of different specific substrate resulting in different readout colours [178,182,183] or via spatially separated pattern shape [184,185] of each enzyme-specific hydrogel.

Such a multiplex approach to monitor several bacteria in parallel would be also needed for monitoring leishmaniasis wounds that can be colonized by various different bacteria [173,186].

Recent work reported on the opportunity to differentiate both Gram-negative bacteria like *E. coli* and Gram-positive bacteria like *S. aureus* from each other via colour-encoded chitosan hydrogels. This detection approach is based on the sensitive and rapid detection of bacterial enzymes in a nanomolar range within one hour. These enzymes are produced by specific bacteria like β-glucuronidase by *E. coli* and α-galactosidase by *S. aureus* [178]. Recently, a colorimetric chitosan-based sensing hydrogel-coated paper to quantify *E. coli* by detection of the enzyme β-glucuronidase was shown to be compatible with a smartphone camera readout. This proves the potential of autonomous enzyme responsive hydrogels towards a laboratory-in-a-phone based point-of-care detection of bacterial contamination, that could be efficiently used also in rural areas of low-income countries, where smartphones are widely available [187].

As a further step, hydrogels are not only able to sense bacterial infections but also to treat such infection upon autonomously triggered release of antibiotics, as theranostic materials [177,188], although the specificity of such smart bandages are highly dependent on the stimulus that triggers the release.

In the case of Leishmaniasis wounds, biocompatible sensory hydrogels have the potential to monitor secondary bacterial infections in an optimal manner and offer additional beneficial characteristics like a good hydration of the wound, their high capacity to take up wound fluid, and their integrability into wound dressings [61].

Hydrogel systems are currently being applied to the treatment of CL (§3.3.3). Further, there are promising approaches to use personalized intelligent patches, e.g. based on smart hydrogels but also integrable bioelectronics for wound healing bandage, in order to both realize *in situ* monitoring of wound status marker and autonomous point of care treatment [189]. However, little has been reported on ‘intelligent’ patches which offer the possibilities to sense and treat CL wounds specifically. They can be engineered to detect bacterial infection and monitor the status of the lesion for proper wound management. For instance, Wang *et al.* developed an injectable chitosan-based hydrogel system doped with pH-responsive bromothymol blue, thermosensitive beta-glycerophosphate and an NIR-absorbing conjugated polymer (PTDBD) [190]. Photothermal PTDBD generated heat to eradicate bacteria upon irradiation of NIR laser inducing a change in pH visualized by a colour change *in situ.* Thus, developing smart sensory hydrogels can prove to be highly beneficial for the detection of secondary bacterial infection, release of anti-leishmanial and antimicrobial agents as well as for visual *in situ* diagnosis in CL.

### 5.2. Non-pharmacological approaches to leishmaniasis

Non-pharmacological approaches to help combat leishmaniasis include modelling studies [191], which are also useful for disease surveillance, analysis of disease dynamics as well as in determining which risk factors and therapeutic approaches are essential in eradicating the disease, smartphone technology [192] and machine learning [193]. Other approaches include public education campaigns that lead to an awareness of the disease and appropriate prevention strategies; control of disease vectors as well as the use of insecticides and mosquito nets in endemic regions.

Leishmaniasis dynamics are complex to model because social-economic factors contribute to disease dynamics. Furthermore, there are a number of *Leishmania* parasites, and the disease is spread by a complex vector process in humans and animals that is influenced by a variety of social and economic factors and through mechanisms that are not fully understood. Nonetheless, there have been a number of mathematical models on the dynamics of leishmaniasis. Discrete time models were first used to study VL dynamics between epidemics based on historical data in Assam, India and the influence of intrinsic and extrinsic factor on the dynamics of the disease [191]. This study showed that in between epidemics, cases of PKDL were probably a disease reservoir, that fuelled future outbreaks. Later, Hasibeder *et al*. [192] developed a compartmental delay-differential equation model to estimate the number of infected sandflies from a single sandfly during disease spread or the reproduction number. The extension of this model to human leishmaniasis was limited because they did not consider asymptomatic cases as a vector source, seasonality of the disease as well as human and vector population variations. Other investigators have since increased the complexity of models of the reproduction number by taking into account zoonotic transmission, seasonality and demographic variations [193–195].

Smartphone technology has the capacity for high-resolution image acquisition, processing and storage, and is widely used in low-resource settings. Cost-effective and easy-to-use smartphone application software may assist health workers in remote settings in detecting the presence and severity of the different forms of leishmaniasis as well as monitoring wound healing. Da Silva and colleagues developed smartphone application software that evaluated the severity of VL based on images acquired by physicians [196]. Over 90% of the 102 health professionals who used their software reported positive expectations and an increase in competency in the treatment of VL. Smartphones were also used to capture images of skin in possible CL cases, which were then relayed to infectious disease experts further from the field and used to assist diagnosis [197]. Despite the high potential of smartphone technology, their use in addressing leishmaniasis remains limited to date.

Machine learning is a form of artificial intelligence that is useful in accomplishing tasks that are easily done by humans but hard to do through conventional computational methods, such as pattern recognition. Through convoluted neural networks, machine learning algorithms can be developed and trained to recognize key features of lower extremity diabetic chronic wounds with success rates of up to 90% [198–200]. Machine learning can also be integrated into smartphones for use by health workers to monitor diabetic chronic wound healing [201]. A similar strategy that integrates smartphone image acquisition capabilities, an image dataset of leishmaniasis wounds and machine learning software can be used to characterize leishmaniasis wounds, monitor wound healing, monitor the success of wound healing intervention strategies and address the lack of experts in low-resource settings. The successful development of this technology would involve collaborative research efforts between clinicians and researchers in biomaterials, biosensors and machine learning.

### 5.3. *In vivo* models to test the nanostrategies in leishmaniasis

Presently, there is no validated animal model for CL and the predictive validity of current animal models is often low due to poor correlation between animal and human disease mechanisms [202]. *In vivo* models for CL should aim to mimic the natural transmission of the disease such as parasite load, the presence of saliva and site of inoculation for accurate representation of disease progression. However, this requires sophisticated facilities and confined laboratories to control the vector.

Hamster, rat species and more commonly inbred mice strains (primary tests), limited dog studies (secondary tests) and non-human primates (tertiary tests) have been used. The objective of dog studies is to understand the pathogenicity of the parasite, since the dog is a natural host of *L. infantum* [203,204]. The BALB/c–*L. major* mice model has been used for testing because of its extreme sensitivity to *L. major* infection and ease of outcome evaluation. The outcome of the infection is directly influenced by the immune responses of the host, and *L. major* infection in BALB/c triggers a strong Th-2 response to leishmanial antigens, leading to rapid lesion growth and generalized infection and, eventually, death. Non-human primate models are used with the aim that their relatedness to humans will lead to a similar mechanism of CL infection and disease progression (table 4).

**Table 4.** Some *Leishmania*–animal models for Old World and New World parasites [202]. +++, Strong evidence for recommendation; ++, More research needed before recommendation.

rodent models			non-human primate models			
BALB/c	humanized mice	Yucatan deer mouse (*Peromyscus yucatanicus*)	vervet monkey (*Chlorocebus pygerythrus*)	Sykes' monkey (*Cercopithecus albogularis*)	rhesus monkey (*Macaca mulatta*)	tufted capuchin (*Cebus apella*)
*L. major*						
Th2/Th1. Visceral disease and death	++ develop cellular components of the human immune system; T, B and NK cells	—	+++ self-healing lesion. IFn-g production by circulating cells do not correlate with cure	++ self-healing lesions	+++ self-healing immune responses similar to humans	—
*L. tropica*						
no lesion, slow growth	—	—	—	—	—	—
*L. mexicana*						
++ large non-healing lesions	—	++ single small lesion	—	—	—	—
*L. amazonensis*						
++ Th2 lesions	—	—	—	—	++ self-healing lesions	++ self-healing lesions Th1/Th2

#### 5.3.1. Importance of the inoculum in model development

The composition and size of inoculum and site of inoculation greatly influences the outcome of infection. Higher doses of inoculum produce larger lesions, which also develop faster. Footpads of mice allow easy measurement of lesion and parasite load [202,205,206]. The base of the tail of rodents and forehead of monkeys are very depicted injection sites [202,207,208]. The ear is used in mice models for inoculation by infected sandflies to simulate natural infection as the needle injections primarily deliver parasites subcutaneously, whereas sandfly introduces the parasite intra-dermally. However, this method requires availability of sandflies and consistent maintenance of infection rates.

*Leishmania* evolves over time and thus it is important to use recent isolates of *Leishmania* from the field for *in vitro* as well as *in vivo* tests [209]. During the course of *in vitro* growth, most laboratories harvest the parasite during the late infective stationary phase of growth and employ peanut agglutinin (PNA) to remove non-metacyclic parasites before inoculation. In rodent models, 1 × 10^6^ metacyclic-enriched parasites are injected in a volume of 50 µl [205–208].

Lastly, it is important to determine in the strategy of the choice of the animal model, the appropriate method to follow the infectious state of the animal. The conventional methods such as microscopy or q-PCR amplification of parasite DNA, are laborious, time consuming and require the euthanasia of large numbers of animals [202,210]. These methods cannot detect the spread of pathogens to unexpected anatomic sites or monitor their space/time progression. The latter can be achieved using reporter molecules which provide a readily measurable phenotype. They are highly sensitive and can be automated for high-throughput quantification. The use of the firefly luciferase reporter molecule in transgenic *Leishmania* species showed promising results in drug evaluation due to its high sensitivity [211]. It is then possible to follow the infectious evolution in the animal using a bioluminescence imaging system without needing to euthanize the animal [210]. This is an ideal refinement for conducting longitudinal studies in a more non-invasive *in vivo* drug screening model and parasite–host interaction studies [212].

## 6. Conclusion

One major complication of CL is secondary microbial infections. Leishmaniasis ulcerated lesions are highly prone to bacterial infections that cause purulent discharges, more damage to the skin tissue, necrosis and inflammation, consequently prolonging the disease and retarding recovery [213]. An effective remedy should be able to efficiently tackle the parasite load and treat any secondary infections occurring simultaneously, thus reducing the drug and financial burden on carers and patients. The challenge also lies in the fact that the wounds are also dependent on the source of the *Leishmania* parasite. A combination of biomaterials, scaffold engineering, biosensors, clinical expertise and machine learning may offer a novel strategic pathway for the well-being of CL patients.

## Data Availability

This article has no additional data.
